# Dehydrocorydaline Accounts the Majority of Anti-Inflammatory Property of Corydalis Rhizoma in Cultured Macrophage

**DOI:** 10.1155/2020/4181696

**Published:** 2020-11-17

**Authors:** Xiangpeng Kong, Zhicong Chen, Yingjie Xia, Etta Y. L. Liu, Haiqin Ren, Cheng Wang, Winnie W. H. Hu, Tina T. X. Dong, Rongbiao Pi, Karl W. K. Tsim

**Affiliations:** ^1^Shenzhen Key Laboratory of Edible and Medicinal Bioresources, HKUST Shenzhen Research Institute, Hi-Tech Park, Shenzhen 518057, China; ^2^Institute of Pharmaceutical and Food Engineering, Chinese Medicine Master Studio of Wang Shimin, Shanxi University of Chinese Medicine, 121 Daxue Road, Yuci District, Jinzhong 030619, China; ^3^Division of Life Science and Center for Chinese Medicine, The Hong Kong University of Science and Technology, Clear Water Bay, Hong Kong, China; ^4^School of Medicine, Sun Yat-Sen University, Guangzhou 510006, China

## Abstract

Corydalis Rhizoma (CR) is a commonly used traditional Chinese medicine for its potency in activating blood circulation and analgesia. In clinic, CR extracts or components are commonly used in the treatment of myocardial ischemia, rheumatism, and dysmenorrhea with different types of inflammation. However, due to different mechanism of pain and inflammation, the anti-inflammatory property of CR has not been fully revealed. Here, the major chromatographic peaks of CR extracts in different extracting solvents were identified, and the anti-inflammatory activities of CR extracts and its major alkaloids were evaluated in LPS-treated macrophages by determining expressions of proinflammatory cytokines, I*κ*B*α* and NF-*κ*B. The most abundant alkaloid in CR extract was dehydrocorydaline, having >50% of total alkaloids. Besides, the anti-inflammatory activities of dehydrocorydaline and its related analogues were demonstrated. The anti-inflammatory roles were revealed in LPS-treated cultured macrophages, including (i) inhibiting proinflammatory cytokines release, for example, TNF-*α*, IL-6; (ii) suppressing mRNA expressions of proinflammatory cytokines; (iii) promoting I*κ*B*α* expression and suppressing activation of NF-*κ*B transcriptional element; and (iv) reducing the nuclear translocation of NF-*κ*B. The results supported that dehydrocorydaline was the major alkaloid in CR extract, which, together with its analogous, accounted the anti-inflammatory property of CR.

## 1. Introduction

Inflammation is an important predisposing factor and one of the leading causes of disease occurrence. Indeed, the excessive inflammatory responses are closely associated with cardiovascular and autoimmune diseases, for example, hyperlipidemia, myocardial ischemia, ischemic stroke, and rheumatoid arthritis [1, 2]. In clinic, the nonsteroidal anti-inflammatory drugs (e.g., aspirin, paracetamol and indomethacin) and glucocorticoids (e.g., beclomethasone dipropionate and budesonide) are commonly used to control inflammatory processes by acting on protein targets in inflammatory signaling [3]. Due to the poor target selectivity, most of these anti-inflammation drugs have shortcomings of side effects, withdrawal rebound, and drug resistance [3, 4]. Traditional Chinese medicine (TCM) has been used in clinic for thousands of years, and many of them show good therapeutic effects in chronic inflammatory diseases. Thus, TCM is playing a comprehensive therapeutic effect in alleviating inflammation-induced clinical symptoms and improving quality of life: the pharmacological properties of TCM are mediated by multicomponents and multitargets [5]. Alkaloid is an important group of secondary metabolite with varieties of biological activities, especially in anti-inflammation and immune-regulation [6]. Historically, there are many classical TCM prescriptions targeting anti-inflammation, for example, Mahuang Xixin Fuzi Tang (described in <<Shanghan Lun>>) in treating allergic rhinitis [7], Huanglian Jiedu Tang (described in <<Waitai Miyao>>) in treating sepsis [8], and Zuojin Wan (described in <<Danxi Xinfa>>) in treating ulcerative diseases [9]. Alkaloids, for example, pseudoephedrine, berberine, and palmatine, in these herbal prescriptions are proposed to alleviate inflammatory response, to reduce tissue damage, and to regulate immune balance [7, 8, 10, 11]. Therefore, this is an efficient way to search anti-inflammatory components with highly effective and low toxicity from TCM.

*Corydalis* Rhizoma (CR; the dried tuber of *Corydalis yanhusuo* (Y. H. Chou and Chun C. Hsu) W. T. Wang ex Z. Y. Su and C. Y. Wu) is a well-known herbal medicine in TCM. Due to the efficacy of activating blood, promoting Qi, and relieving pain, CR has been widely used in treating varieties of pain for years, for example, stomachache, cardialgia, and dysmenorrhea. In clinical application, CR is usually used by combining with other herbal medicines, for example, herbal mixtures of Yingshen San (described in <<Weishi Jiacang Fang>>), Ersheng San (described in <<Qixiao Liangfang>>), and Shixiao San (described in <<Taiping Huimin Hejiju Fang>>) in treating abdominal cold pain, colic pain, and blood stasis pain [12–14]. In addition, CR extracts had good analgesic and anti-inflammatory effects in mouse twisting and foot swelling experiments [15]. In parallel, dehydrocorydaline, an alkaloid in CR, was shown to have antiallergic effect in collagen type I-IV allergic models [16], while rotundinum (tetrahydropalmatine), isocorypalmine, and corydalmine showed analgesic activity via dopamine, NMDA, and mGlu1/5 receptors [17–20]. By spectrum-effect relationship analysis, the CR alkaloids, that is, coptisine, berberine, palmatine, and dehydrocorydaline, were proposed to be closely related to anti-inflammatory potency of CR extracts [21]. Due to different physiological mechanisms of pain and inflammation, the mechanistic studies of CR, as well as its active components, on anti-inflammation have not been fully revealed. In general, CR contains 0.5 to 1% of total alkaloids [22, 23]. Indeed, the protoberberine quaternary amine alkaloids (e.g., columbamine, coptisine, palmatine, berberine, and dehydrocorydaline), hydrogenated tertiary amine alkaloids (e.g., isocorypalmine, stylopine, rotundinum, canadine, and corydaline), proopioid protoberberine alkaloids (e.g., protopine and allocryptopine), and aphis alkaloids (e.g., glaucine) are the main parent structure of alkaloids being found in CR [22, 23]. Here, the anti-inflammatory activities of dehydrocorydaline, corydaline, and corydalmine were chosen for further study.

## 2. Materials and Methods

### 2.1. Chemicals and Herbal Extract Preparation

Berberine (Lot # SLBT0096, purity 99%) was purchased from Sigma- Aldrich (St. Louis, MO); palmatine (Lot S0805239, purity 99%) was purchased from Chengdu Ruifensi Biotechnology (Chengdu, China); Columbamine (Lot DST190528-009, purity 99%), coptisine (Lot DST170711-003, purity 99%), glaucine (Lot DST190720-176, purity 99%), dehydrocorydaline (Lot DST190422-025, purity 99%), rotundinum (Lot DST190718-331, purity 99%), canadine (Lot DST180127-065, purity 99%), corydaline (Lot DST190701-102, purity 99%), stylopine (Lot DST180127-158, purity 99%), allocryptopine (Lot DST190322-091, purity 99%), corydalmine (Lot DST190602-726, purity 99%), and isocorypalmine (Lot DST190612-157, purity 99%) were purchased from Chengdu DeSiTe Biological Technology (Chengdu, China).

CR is the tuber of *C. yanhusuo* from Zhejiang, China. The authentication of CR herb was performed by Professor Xiangping Pei from Shanxi University of Chinese Medicine. The CR herb was weighed appropriately, and each of them was extracted twice (1st, 1 : 10 w/v, 40 min; 2nd, 1 : 8 w/v, 30 min) by reflux with 0%, 25%, 50%, 75%, and 100% ethanol volume fraction, and the twice filtrates were combined, concentrated to sticky state, and dried at 60°C. The herbal extracts of CR were weighed accurately and dissolved to stock solution with concentration of 40 mg/mL by DMSO. The standards of dehydrocorydaline, corydaline, and corydalmine were weighed accurately and dissolved to stock solution with concentrations of 50 mM by DMSO, respectively. The different concentrations of these alkaloids were prepared for evaluation of cell viability and anti-inflammatory activity by diluting from initial stocks.

### 2.2. Determination of Alkaloids

The herbal extracts of CR were weighed accurately and dissolved by 50% methanol with concentration of 2.5 mg/mL. The dissolved extract was filtered by 0.22 *μ*m Millipore filter, and subsequently the filtrate was collected for HPLC determination. The standards of columbamine, coptisine, palmatine, berberine, dehydrocorydaline, glaucine, rotundinum, corydaline, and stylopine were weighed accurately and dissolved to stock solution at 1 mg/mL by methanol. Different volumes of columbamine, coptisine, palmatine, berberine, dehydrocorydaline, glaucine, rotundinum, corydaline, and stylopine stocks were mixed to prepare the stock solution of alkaloids, with final concentrations of 96.00, 93.33, 126.98, 47.62, 349.21, 44.44, 96.00, 132.06, and 63.56 *μ*g/mL, respectively. After that, the stock solutions of alkaloids in CR were diluted to series of working standards by methanol. The chromatographic analysis was performed by Waters 2695 HPLC with PDA detector. The separation of CR extract was achieved on an Innoval C18 column (4.6 × 250 mm, 5 *µ*m) with a constant flow rate of 1.0 mL/min at 25°C. The mobile phase of CR was composed of MeCN (*A*) and water (*B*) containing 0.1% phosphoric acid and 0.22% triethylamine, using a gradient elution of 20–22% at 0–10 min, 22–30% at 10–30 min, and 30–80% at 30–60 min. The analyses were detected at 280 nm. The injection volume was set at 10 *µ*L.

### 2.3. Cell Culture

Murine macrophage RAW 264.7 cell line was obtained from American Type Culture Collection (ATCC account number TIB-71; Manassas, VA). The cells were cultured in Dulbecco's modified Eagles medium (DMEM; Life Technologies, Carlsbad, CA) containing 10% fatal bovine serum (FBS, A Biochemical Company, Shanghai, China), 100 IU/mL penicillin (Sigma, St. Louis, MO), and 100 *μ*g/mL of streptomycin (Sigma). The cells were cultured in 75 cm^2^ plastic plate in a humidified atmosphere with 5% CO_2_ at 37°C and supplied with fresh DMEM culture medium every other day. After 80% confluence, the cells were scraped with a scraper, collected by centrifugation, resuspended in fresh DMEM culture medium, and seeded into new cell plate for inflammatory activity assay. Reagents for cell cultures were purchased from Shanghai Univ Biotechnology (Shanghai, China).

### 2.4. Cell Viability Assay

The cell viability of CR extracts and alkaloids was measured by colorimetric 3-(4,5-dimethylthioazol-2-yl)-2,5-diphenyltetrazolium bromide (MTT) assay. In brief, the cultured RAW 264.7 cells were seeded in 96-well plates with 5 × 10^4^ cells/well and treated with different concentrations of CR extracts and alkaloids for 24 hours. Then, the culture medium was discarded, and the cultured cell wells were added with 100 *μ*L MTT solution (0.5 mg/mL) for 4 hours followed by adding 150 *μ*L DMSO. The cell viability was determined by measuring the absorbance at 495 nm by calculated as the percentage of absorbance value of negative control (without drug treatment), where the absorbance value was set as 100%.

### 2.5. Measurement of Proinflammatory Cytokines

The proinflammatory cytokines (TNF-*α* and IL-6) secreted by RAW 264.7 cells were measured by Mouse/Rat Valukine ELISA Kit (Shanghai Univ Biotechnology) according to the manufacturer's guidelines. The cells were seeded in 24-well plate with 4 × 10^5^ cells/well for sticking cultivation. The cultured RAW 264.7 cells were pretreated with different concentrations of CR extracts (5, 10, 15, and 20 mg/mL), dehydrocorydaline (2.5, 5, 10, and 20 *μ*M), corydaline (10, 15, 30, and 60 *μ*M), and corydalmine (15, 30, 60, and 90 *μ*M) for 2 hours and then treated with lipopolysaccharide (LPS; Solarbio Life Sciences, Beijing, China) at 20 ng/mL for 6 hours to induce cellular inflammation. The positive control cells were pretreated with dexamethasone (Dex; Solarbio Life Sciences) at 20 *μ*M and treated with LPS; the negative control cells were not treated with drug and LPS. After that, the cultured medium was collected to measure the concentrations of proinflammatory cytokines (TNF-*α* and IL-6) by measuring the absorbance difference between 450 and 570 nm using Thermo Scientific Multiscan FC. Meanwhile, the cells were washed twice with ice-cold phosphate buffered saline (PBS), and the cell plate was frozen at −80°C refrigerator for quantitative real-time PCR or western blot analysis. Protein concentration was measured by Bio-Rad Protein Assay Dye Reagent (Hercules, CA).

### 2.6. Quantitative Real-Time PCR

The mRNA expressions of TNF-*α*, IL-6, and IL-1*β* in cultured RAW 264.7 cells, treated with CR extracts (2.5, 5, 10, 15 and 20 mg/mL), dehydrocorydaline (2.5, 5, 10 and 20 *μ*M), corydaline (10, 15, 30 and 60 *μ*M), corydalmine (15, 30, 60 and 90 *μ*M), and dexamethasone (20 *μ*M), were detected by quantitative real-time PCR. The total RNA of RAW 264.7 cells in 24-well plate was isolated by RNA simple Total RNA kit (Tiangen Biotech, Beijing, China) and reversed transcribed into first-strand cDNAs synthesis by FastKing RT kit (World's Foregene Biotech, Chengdu, China) according to the manufacturer's specification. Real-time PCR was employed here by SuperReal PreMix Plus kit (World's Foregene Biotech) according to the manufacturer's instruction. The primers were as follows: 5′-AGT GAC AAG CCT GTA GCC-3′ (S) and 5′-AGG TTG ACT TTC TCC TGG-3′(AS) for murine TNF-*α* (251 bp; NM_013693); 5′-GGA GTA CCA TAG CTACCT GG-3′ (S) and 5′-CTA GGT TTG CCG AGT AGA TC-3′ (AS) for murine IL-6 (283 bp; NM_031168); 5′-AAA TAC CTG TGG CCT TG-3′ (sense primer, S) and 5′-TTA GGA AGA CAC GGA TTC-3′ (antisense primer, AS) for murine IL-1*β* (296 bp; NM_008361). Glyceraldehyde 3-phosphate dehydrogenase (GAPDH) was used as an internal control, and its primer sequences were 5′-AAC GGA TTT GGC CGT ATT GG-3′ (S) and 5′-CTT CCC GTT CAG CTC TGG G-3′ (AS) (657 bp; NR_0215885). The SYBR green signal was detected by Agilent Technologies Stratagene Mx3000P (Santa Clara, CA). Each sample was run in triplicate. Transcript levels were quantified by the *ΔΔ*Ct value method, where the values of target genes were normalized by the GAPDH in the same sample at first before comparison.

### 2.7. Western Blot Assay

The activation of I*κ*B*α* (∼39 kDa) in cultured RAW 264.7 cells treated with dehydrocorydaline (5, 10 and 20 *μ*M), corydaline (15, 30 and 60 *μ*M), corydalmine (30, 60 and 90 *μ*M), and dexamethasone (20 *μ*M) were determined by western blot assay. The cells were harvested with lysis buffer, shaken for 15 min, followed by centrifugation at 13,200 rpm for 15 min at 4°C. The protein content in supernatant was determined by Bradford assay with bovine serum albumin (Solarbio, Lot 630P055), as standard. Samples were adjusted to the same amount of total protein. The homogenates were lysed with 2 × loading buffer and boiled for 5 min before being transferred to the 10% gel electrophoresis. The gel was run in electrophoresis buffer at 60 V for 30 min in stacking gel and at 90 V for 60 min in resolving gel. After electrophoresis separation, the proteins were moved from SDS-PAGE to a nitrocellulose membrane, using a Mini Trans-Blot Cell at 70 V, 0.2 A for 1 hour in 1 × transfer buffer. The membrane was stained with *Ponceau S* to affirm the transfer and equal addition of the samples. After that, the membrane was blocked with 5% skim milk powder in TBS-*T* (Tris-buffered saline and Tween 20) for 1 hour at room temperature and then incubated with primary anti-I*κ*B*α* antibody (1 : 5,000, Santa Cruz Biotechnology, Santa Cruz, CA) for 12 hours at 4°C. After washing with TBS-T, HRP-conjugated anti-rabbit secondary antibody (ImmunoWay Biotechnology Company, Plano, TX) at 1 : 5,000 dilution was added and incubated for 1 hour at room temperature. The immune complexes were scanned using the ECL method (A Biochemical Company). The expression level of protein was calculated using *α*-tubulin as an internal control.

### 2.8. DNA Transfection and Luciferase Assay

The vector, pGL4.32 [luc2P/NF-*κ*BRE/Hygro], contains five copies of an NF-*κ*B response element (NF-*κ*B, 5′-GGG AAT TTC CG-3′) that drives transcription of the luciferase reporter gene luc2P (*Photinus pyralis*) (Promega Corporation, Madison, WI). Transient transfection of RAW 264.7 cells in 24-well plate with the cDNA vector was performed with jetPRIME® reagent, according to the manufacturer's instruction. The transfection efficiency was over 50% in RAW cell culture, as determined by another control plasmid having a *β*-galactosidase gene under a cytomegalovirus enhancer promoter. The luciferase assay was performed by a commercial kit (Tropix Inc., Bedford, MA). In brief, the transfected cells in 24-well plate were treated with different concentrations of dehydrocorydaline (2.5, 5, 10, and 20 *μ*M), corydaline (10, 15, 30, and 60 *μ*M), corydalmine (15, 30, 60, and 90 *μ*M), and dexamethasone (20 *μ*M), followed by treated with LPS (20 ng/mL) referring to steps in “measurement of proinflammatory cytokines.” The cells were washed twice with ice-cold PBS after the medium was aspirated and lysed by 100 mM potassium phosphate buffer (pH 7.8) containing 0.2% Triton X-100 and 1 mM dithiothreitol (DTT) at 4°C. The resuspended lysate was centrifuged at 13,200 rpm 20 min; then the supernatant was collected and used to perform luciferase assay (Tropix). The activity was expressed as absorbance (up to 560 nm) per mg of protein.

### 2.9. Immunofluorescent Staining

The cultured macrophage cells were placed on glass coverslips and treated with dehydrocorydaline, corydaline, and corydalmine at concentrations of 20, 60, and 90 *µ*M, respectively. In parallel, the positive control cells (treated with dexamethasone and LPS) and negative control cells (not treated with drug and LPS) were performed. After being washed with PBS, the cells were fixed with 4% formaldehyde for 15 min. Then, the cells were blocked by 0.5% BSA with 0.2% Triton *X*-100 for 1 hour at room temperature. The cells were incubated with anti-NF-*κ*B antibody (Abcam) at 1 : 500 for overnight at 4°C, followed by the Alexa Fluor 555-conjugated anti-rabbit antibody (Sigma) together with 4′,6-diamidino-2-phenylindole (DAPI) staining (5 mg/mL) for 2 hours at room temperature (Sigma). After being washed, the samples were then examined by a Leica SP8 confocal microscope.

### 2.10. Statistical Analysis

Statistical tests were done by DPS software [24]. Data were expressed as Mean ± SEM of three independent experiments, with triplicate at each experiment. Comparisons of the means for untreated control cells and treated cells were analyzed using Student's *t*-test. Significant values were indicated by ^*∗*^*p* < 0.05 and ^*∗∗*^*p* < 0.01.

## 3. Results

### 3.1. Determination of Alkaloids in CR Extracts

**Figure 1:**
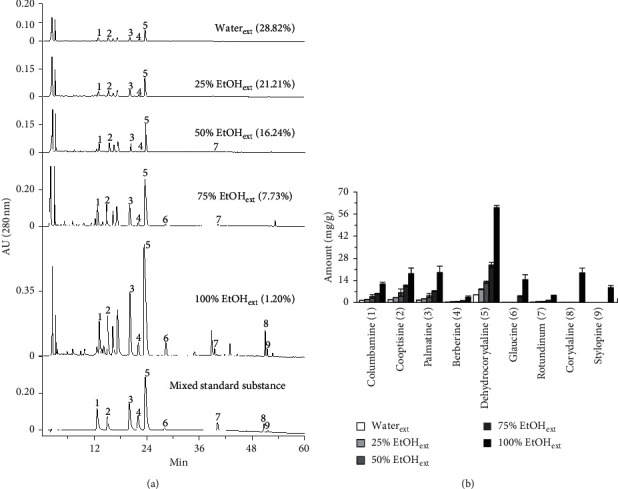
The HPLC chromatograms and alkaloid determination of CR extracts in different extracting solvents. (a) Typical profiles and yields of CR extracts for each solvent, and the mixed standard substance chromatogram were shown here. The main alkaloids of CR extracts were identified (number as in (b)) by known chemical standards. (b) The alkaloids, determined in CR extracts, were shown. The identification numbers of alkaloids in CR extracts are labeled in HPLC chromatograms, as shown. Values are in mean ± SEM, *n* = 3.

**Figure 2:**
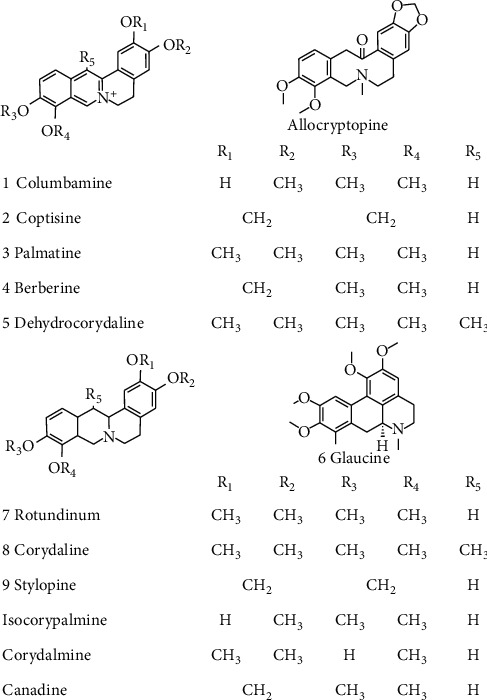
The structures of CR alkaloids. The structures of CR alkaloids are numbered and listed according to nuclear structural characters from 1 to 9, as being detected in the chromatogram, as in Figure 1. Known or related alkaloids in CR, but not being detected here, are also presented.

Alkaloids are the main components in CR extracts. The HPLC chromatograms of CR extracts by using different solvents of having varied amount of ethanol are shown in Figure 1(a). The major alkaloids, that is, columbamine, coptisine, palmatine, berberine, dehydrocorydaline, glaucine, rotundinum, corydaline, and stylopine, were identified in the fingerprints, and their content in different extracts was determined and shown in Figure 1(b). The structures of major CR alkaloids are shown in Figure 2. Besides, the closely related alkaloids previously found in CR, for example, allocryptopine, isocorypalmine, corydalmine, and canadine, were also presented; however, these alkaloids were not found in our current analysis. The amounts of alkaloids in CR crude drug were determined, and the linear relationships having *R*^2^ of each component > 0.999 are shown in Table 1. The alkaloid content of CR extracts increased obviously with ethanol concentration in the extracting solvent. Maximal amount of alkaloid was revealed in solvent of 100% ethanol, that is, about 140 mg per gram of extract. In parallel, the peaks showing amount of alkaloids in CR were increased with an increase of ethanol fraction in extracting solvent (Figure 1(a)). The yields of CR extractives however decreased when the ethanol fraction increased (Figure 1(a)). Dehydrocorydaline was the highest abundant alkaloid in CR extracts, nearly accounting for 50% of total alkaloid content. Corydaline, an analogue of dehydrocorydaline, also occurred in high amount in CR ethanol extract.

### 3.2. CR Extracts Inhibit Expression of Proinflammatory Cytokines

Before measurement for anti-inflammatory activity, the effect of CR extracts on cell viability in RAW 264.7 cells was firstly determined by MTT assay. By comparing to the control (only DMSO treatment), CR extracts of water and 25%, 50%, and 75% ethanol fraction showed no significant effect on RAW 264.7 cell viability at concentration not more than 30 *μ*g/mL (Figure S1). At concentration not more than 20 *μ*g/mL, the CR extract of 100% ethanol showed no significant effect on RAW 264.7 cell viability. To compare anti-inflammatory activity of CR extracts, different concentrations of CR extracts with no cytotoxicity were chosen for further experiments. Similarly, different concentrations of CR alkaloids, for example, dehydrocorydaline, corydaline, and corydalmine, were applied onto cultured RAW 264.7 cells for 24 hours, followed by MTT cell viability assay. Thus, the nontoxic doses of these extracts or alkaloids were used in the following culture experiments.

**Figure 3:**
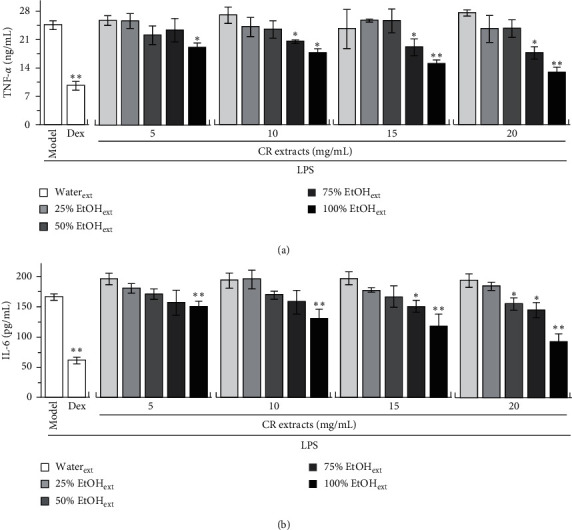
CR extracts inhibit protein expressions of proinflammatory cytokines. Cultured RAW 264.7 cells in 24-well plates were treated with different doses of CR extracts by water and different fractions of ethanol (25%, 50%, 75%, and 100%) for 2 hours, followed by added LPS (20 ng/mL) and cultured for further 6 hours. The doses of CR extracts from 5 to 20 mg/mL were chosen as noncytotoxicity according to their cell viability in Supplement Figure 1. The cultured medium was collected and tested for levels of proinflammatory cytokines by ELISA. Data are expressed as mean ± SEM, *n* = 3, each with triplicate samples. ^*∗*^*p* < 0.05 and ^*∗∗*^*p* < 0.01, compared to model control (LPS but no CR extracts).

RAW 264.7 macrophage is often used as inflammatory cell model, and the release of proinflammatory cytokines, for example, TNF-*α*, IL-6, and IL-1*β*, could be stimulated markedly by applied LPS. Cultured macrophages were incubated with CR extracts in 24-well plates, followed by adding LPS (20 ng/mL). The proinflammatory cytokines in culture medium were measured by ELISA. As shown in Figure 3, the inhibitory activities of CR extracts from 5 to 20 mg/mL on the LPS-induced expression of proinflammatory cytokines increased with an increase of alkaloid content, while CR extracts of water and 25% ethanol showed no inhibition on cytokine expression; CR extracts of 50% and 75% ethanol showed weak inhibition on cytokine expression. The CR extract of 100% ethanol showed good inhibition on the LPS-induced expressions of TNF-*α* and IL-6 (Figure 3). The cytokine suppression was increased in accordance with the amount of ethanol in extracting solvent, as well as dose of applied herbal extracts. Dexamethasone was used as a positive control.

**Figure 4:**
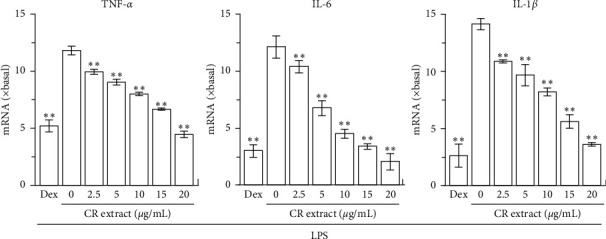
CR extract inhibits transcription of proinflammatory cytokines. Cultured RAW 264.7 cells in 24-well plates were treated with the different doses of CR extract of 100% ethanol, as well as LPS (20 ng/mL), as described in Figure 3. After the treatment, the cells were harvested for measurement of TNF-*α*, IL-6, and IL-1*β* mRNAs by real-time PCR. Data are expressed as fold of change to basal (no drug), in mean ± SEM, *n* = 3, each with triplicate samples. ^*∗*^*p* < 0.05 and ^*∗∗*^*p* < 0.01, compared to model control (LPS but no CR extract).

The mRNA expressions of proinflammatory cytokines in RAW 264.7 cells, determined by quantitative real-time PCR, were markedly induced by LPS (Figure 4). In LPS-treated cultures, application of ethanolic CR extract suppressed the expressions of mRNAs encoding TNF-*α*, IL-6, and IL-1*β*, which were in dose-dependent manners. The ethanolic CR extract at 20 mg/mL fully suppressed the inflammation, triggered by applied LPS: this robust suppression was similar to that of dexamethasone (Figure 4). As seen from the alkaloid composition of CR extract, the quaternary protoberberine alkaloids were the main components in CR. Besides, dehydrocorydaline was the highest abundant component among CR quaternary protoberberine alkaloids. Therefore, it was strongly indicated that dehydrocorydaline and other quaternary protoberberine alkaloids could be the major player in anti-inflammatory property of CR.

### 3.3. Dehydrocorydaline and Its Analogues Inhibit Expression of Proinflammatory Cytokines

The CR alkaloids, for example, columbamine, palmatine, glaucine, and berberine, are known to have anti-inflammatory activity [10, 11, 25]. Dehydrocorydaline and corydaline are the most abundant alkaloid in CR. However, the anti-inflammatory activity or mechanism of dehydrocorydaline and corydaline, as well as its closely related analogues corydalmine, was less studied. Although corydalmine was not detected in our CR extracts, this alkaloid was reported in CR extracts. Thus, dehydrocorydaline, corydaline, and corydalmine were selected and tested for inhibition of proinflammatory cytokine expression. The cell viability of CR alkaloid was determined by MTT assay. As shown in Figure S1, dehydrocorydaline, corydaline, and corydalmine showed no significant effect on macrophage at concentrations of 20, 90, and 120 *μ*M, respectively. Therefore, the maximal concentration of alkaloids in the following experiments should not be higher than their toxic concentration.

**Figure 5:**
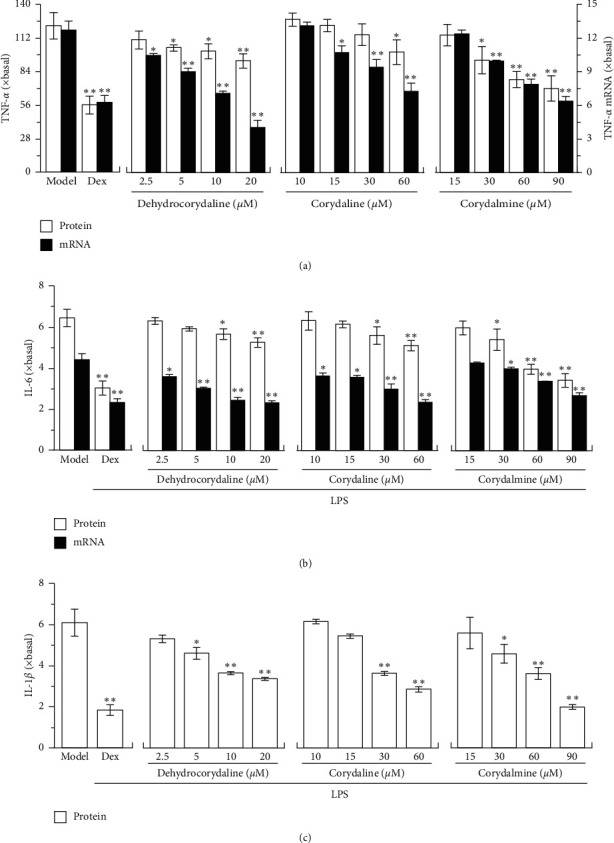
CR alkaloids inhibit transcription of proinflammatory cytokines. Cultured RAW 264.7 cells in 24-well plates were treated with different doses of dehydrocorydaline, corydaline, and corydalmine, as described in Figure 3. The cultured medium was collected and tested for protein levels of proinflammatory cytokines by ELISA, and the cells were harvested for mRNA expression of proinflammatory cytokines by real-time PCR. (a) The protein (left vertical axis) and mRNA (right vertical axis) levels of TNF-*α*. (b) The protein and mRNA levels of IL-6. (c) The mRNA level of IL-1*β*. Data are expressed as fold of change to basal (no drug), in mean ± SEM, *n* = 3, each with triplicate samples. ^*∗*^*p* < 0.05 and ^*∗∗*^*p* < 0.01, compared to model control (LPS but no CR alkaloid).

Cultured macrophages were incubated with the alkaloids in 24-well plates followed by using LPS to activate cell inflammation. The noncytotoxic dosages of dehydrocorydaline, corydaline, and corydalmine were applied in LPS-treated macrophages. In LPS-treated cultures, the applied alkaloids suppressed the protein and mRNA expressions of TNF-*α* in dose-dependent manners (Figure 5(a)). Dehydrocorydaline, the highest abundant alkaloid in CR extracts, showed robust effect of anti-inflammation. The expression of IL-6 in LPS-treated cultures was downregulated by dehydrocorydaline, corydaline, and corydalmine: this suppression was revealed in both mRNA and protein levels of IL-6 (Figure 5(b)). Moreover, the mRNA expression of IL-1*β* in LPS-treated macrophages was suppressed by applied dehydrocorydaline, corydaline, and corydalmine (Figure 5(c)). Therefore, dehydrocorydaline, corydaline, and corydalmine suppressed the inflammation at different magnitudes, that is, reducing production of proinflammatory cytokines. Besides, the expression of mRNA was more sensitive to that of the protein in responding to alkaloid treatment.

### 3.4. Dehydrocorydaline and Its Analogues Regulate I*κ*B*α* and NF-*κ*B

**Figure 6:**
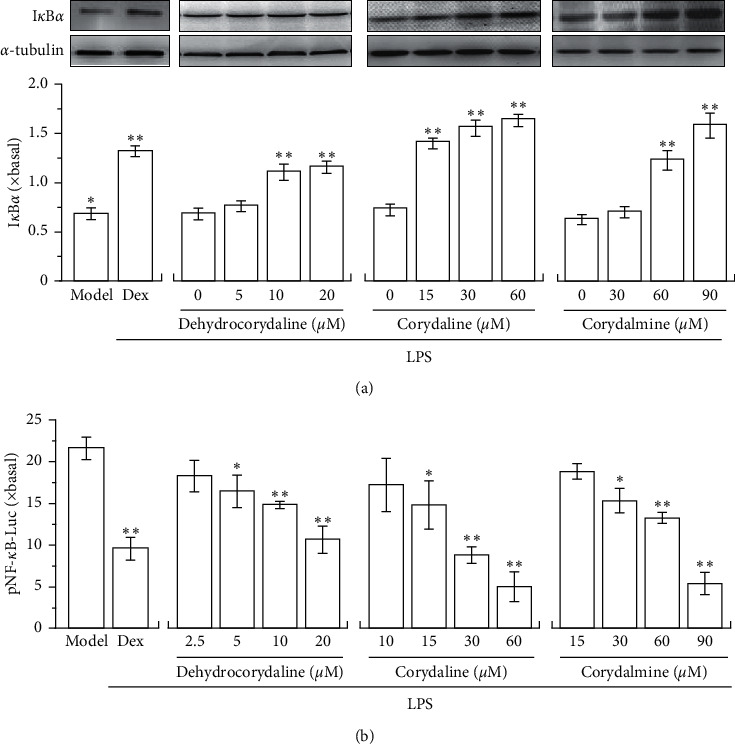
CR alkaloids regulate I*κ*B*α* and NF-*κ*B. (a) Cultured RAW 264.7 cells in 24-well plates were treated with different doses of dehydrocorydaline, corydaline, and corydalmine, as described in Figure 3. The cells were harvested for expression of I*κ*B*α* by western blot (upper panel). The expression of *α*-tubulin served as a loading control. Quantitation of protein bands was shown (lower panel). (b) Cultured 264.7 cells in 24-well plates were transiently transfected with pNF-*κ*B-luc for 24 hours; after that, they were pretreated with alkaloids as in (a). Then, the cell lysates were collected for luciferase assay. Data are expressed as fold of change to basal (no drug), in mean ± SEM, *n* = 3, each with triplicate samples.^*∗*^*p* < 0.05 and ^*∗∗*^*p* < 0.01, compared to model control (LPS but no alkaloid).

Nuclear factor-kappa B (NF-*κ*B) is an important transcription factor playing a crucial role in promoting expression of proinflammatory cytokine. Normally, NF-*κ*B is a deactivated state by masking with I*κ*B*α* in cytosol [26], which is activated by degrading I*κ*B*α* when stimulated by LPS. In order to investigate the anti-inflammatory pathway of alkaloids in cultured RAW 264.7 cells, the expressions of I*κ*B*α* protein and pNF-*κ*B-Luc were determined by western blot and luciferase assay. In LPS-treated cultures, application of dehydrocorydaline, corydaline, and corydalmine promoted the expression of I*κ*B*α* in a dose-dependent manner: the maximal induction was robust, as good as that of dexamethasone (Figure 6(a)). In this activation, corydaline showed the best result. In pNF-*κ*B-Luc transfected cultures, the applied LPS induced the luciferase activity by ∼20 folds. The induction was markedly suppressed by dehydrocorydaline, corydaline, and corydalmine in dose-dependent manner (Figure 6(b)). Here, dehydrocorydaline, corydaline, and corydalmine showed better regulation in suppressing pNF-*κ*B-Luc activity in RAW 264.7 cells.

**Figure 7:**
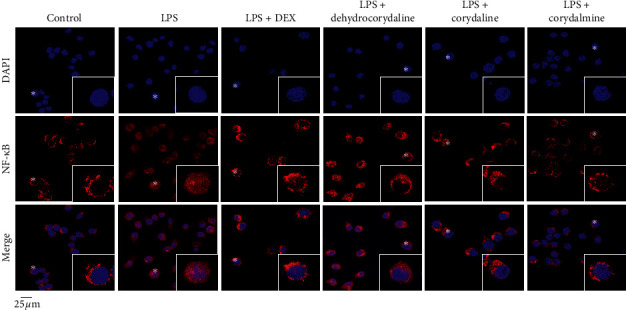
CR alkaloids suppress LPS-induced translocation of NF-*κ*B. Cultured RAW 264.7 cells were treated with dehydrocorydaline, corydaline, and corydalmine at concentrations of 20, 60, and 90 *µ*M, respectively, as described in Figure 3. Immunofluorescence staining was applied to observe the localization of NF-*κ*B p65 by antibody (red fluorescence) and in nuclei staining by DAPI (blue fluorescence) in RAW 264.7 cells. Asterisks indicated nuclei with NF-*κ*B staining, and also the enlarged cell was in the bottom right corner.

The translocation of NF-*κ*B into nucleus contributes to inflammatory responses by upregulating transcription of key inflammatory genes. To further confirm anti-inflammatory mechanism of dehydrocorydaline, corydaline, and corydalmine on NF-*κ*B signaling, the nuclear translocation of NF-*κ*B in drug-treated RAW 264.7 cells was determined by immunofluorescence staining (Figure 7). The recognition of NF-*κ*B and nucleus was identified by red and blue color, respectively. Compared to control cells (not treated with drug and LPS), the nuclear accumulation of NF-*κ*B was induced by ∼2 folds in LPS-treated cultures. However, the application of dehydrocorydaline, corydaline, and corydalmine attenuated the nuclear translocation of NF-*κ*B obviously in LPS-treated cultures, that is, reducing the nuclear accumulation of NF-*κ*B by over 60%. Thus, dehydrocorydaline, corydaline, and corydalmine showed robust effect in attenuating nuclear accumulation of NF-*κ*B in RAW 264.7 cells.

## 4. Discussion

CR is a common TCM being used in treating varieties of pain. Except the usage in relieving pain, the herbal extracts of CR, or its alkaloids, are commonly used in treatment of myocardial ischemia, rheumatism, and dysmenorrhea, and most of these medical problems are involving different types of inflammation [27–29]. Alkaloids are commonly found in herbal medicines that are used for anti-inflammation. For example, berberine, originated from *Coptis chinensis* Franch. and *Phellodendron chinense* Schneid., inhibited expression of proinflammatory cytokine by modulating Sirt1/NF-*κ*B signaling [11]. Palmatine and columbamine, originated from *Tinospora sagittata* (Oliv.) Gagnep, inhibited production of nitric oxide and activation of NF-*κ*B [10]. Glaucine, isolated from *Glaucium flavum* Crantz. (Papaveraceae), reduced proinflammatory cytokine, and increased anti-inflammatory cytokine (IL-10) by Toll-like receptor-mediated signaling [25]. Stylopine, isolated from *Corydalis impatiens*, reduced the production of proinflammatory cytokine by inhibiting NF-*κ*B and MAPK signaling [30]. In line with other alkaloid-containing herbs, CR has been shown to reduce the acetic acid-induced mice writhing and relieve ear swelling and neuropathic pain [15], while rotundinum, the quality chemical marker of CR (described in <<Pharmacopoeia of P.R. China>>, 2020), is able to inhibit the secretion of proinflammatory cytokine by blocking MAPK phosphorylation [31]. Dehydrocorydaline, corydaline, corydalmine, and isocorypalmine are the representing alkaloids in analgesic effect of CR usage [17, 19, 20]; however, due to different mechanism of pain and inflammation, the signaling of anti-inflammatory property of these alkaloids has not been fully revealed.

The anti-inflammatory potency of CR extracts with high amount of alkaloids was stronger than that of CR extracts with low amount of alkaloids, that is, the CR extract of 100% ethanol fraction significantly inhibited the expression of proinflammatory cytokine with a good dose-effect relationship. In line with this notion, our current results showed that the abundant alkaloid (dehydrocorydaline and corydaline) demonstrated anti-inflammatory activity in LPS-induced macrophages. Besides, as seen from Figures 1 and 2, the protoberberine alkaloids with quaternary nuclear structure were the main components of CR, in which dehydrocorydaline was the highest abundant component, accounting for about half of total alkaloid. Interestingly, the potency of dehydrocorydaline in suppressing expression of proinflammatory cytokines was stronger than corydaline and corydalmine. Therefore, similar to the structure-activity relationship of protoberberine alkaloids from *Corydalis* species on acetylcholinesterase (AChE) inhibitory activity [32, 33], it is speculated that the quaternary nuclear structure with positively charged nitrogen atom (N+) could enhance the anti-inflammatory activity of protoberberine alkaloids. Besides the protoberberine scaffold, the methylenedioxy substitutions at R1, R2, R3, R4, and R5 positions of benzene ring are also strongly correlated with the anti-inflammatory activity of protoberberine alkaloids, which however need to be studied further.

AChE is an enzyme to hydrolyze acetylcholine in cholinergic synapses, and *α*7nAChR is an important inflammation-related receptor in many kinds of immune cells [34]. These two cholinergic molecules have been known to play important role in “cholinergic anti-inflammatory pathway (CAP)” by regulating cholinergic anti-inflammatory response [35], which includes the release of proinflammatory cytokines (TNF-*α*, IL-6, and IL-1*β*) in macrophage by controlling the level of acetylcholine and activation of *α*7nAChR. In addition, recent studies have shown that the inflammation, induced by LPS, could upregulate expression of AChE in macrophage [36]. In parallel, AChE-targeting microRNA-132 could attenuate inflammation by reducing AChE level in immune cells [37]. In addition, the regulation of AChE expression could be triggered by cyclic AMP responding element acting on the AChE gene promoter, and this regulation could be induced by phytochemicals [38]. Many alkaloids, for example, huperzine A from *Huperzia serrata*, fangchinoline from *Stephania tetrandra,* and coptisine from *C. chinensis*, showed good inhibitory activity on AChE [39]. Here, most of the CR alkaloids showed AChE inhibitory activity at different magnitudes [32]. Moreover, there is a positive correlation between AChE and inflammation inhibition, that is, quaternary ammonium alkaloids (e.g., berberine, palmatine, dehydrocorydaline, and columbamine), having strong AChE inhibitory and anti-inflammatory effects compared to their hydrogenated tertiary amine alkaloids (e.g., canadine, rotundinum, corydaline, and isocorypalmine). Thus, the anti-inflammatory function of CR alkaloids could be, at least partly, contributed by its AChE inhibition, which needs to be studied further.

Alkaloids are a group of important naturally components in medicinal herbs. As the major active component, the amount of alkaloid in CR could account for 0.5–1% of total weight [22, 23]. As calculated from yields of CR extracts and alkaloid content in CR extracts, ethanol should be considered as the best solvent in total extractive yield and total alkaloid, which is much better than that of water [40]. Decoction, powder, pill, elixir, and ointment are common forms of TCM preparations during clinical application. From over 1,000 CR-contained herbal prescriptions described in “Dictionary of TCM Prescriptions,” the frequency of CR used in crude powder is 2.6 times higher than that of CR used in a form of water decoction. Besides, among 256 CR-contained Chinese patent medicine prescriptions in “Compilation of National Proprietary Chinese Medicine Standards,” “Ministry of Health Drug Standards, Chinese Medicine Formulation,” and “Pharmacopoeia of P.R. China,” the frequency of CR being used in form of crude power or ethanol extract is about 2 times higher than that of CR being used in water extract. Therefore, the historical usage of CR, in powder or in ethanol extract, strongly supports the current results that alkaloids could be the active components of this herb.

## 5. Conclusions

Alkaloids are the major components in CR extracts, and the anti-inflammatory property of CR extracts increased in parallel to the amount of alkaloid in CR extracts. Besides, the highest abundant, quality chemical, and/or analgesic alkaloids, including dehydrocorydaline, corydaline, and corydalmine, demonstrated good anti-inflammatory activity in reducing the release of proinflammatory cytokine and suppressing the gene expression of proinflammatory cytokine. In addition, their application promoted I*κ*B*α* expression, suppressed activation of NF-*κ*B transcriptional element, and reduced the nuclear accumulation of NF-*κ*B. Therefore, the results supported that the alkaloids were the major anti-inflammatory active compounds in CR extracts, and the anti-inflammation pathway of dehydrocorydaline, corydaline, and corydalmine was firstly revealed.

## Figures and Tables

**Table 1:** The linearity curves and content of main alkaloids in CR crude drug.

Alkaloids	Linear equation	*R* ^2^	Linear range (*μ*g/mL)	Content (mg/g)
Columbamine	*y* = 43952*x* − 58219	0.9993	1.50 – 96.00	0.4644 ± 0.0335
Coptisine	*y* = 29249*x* − 35590	0.9994	1.45 – 93.33	0.6500 ± 0.0037
Palmatine	*y* = 40983*x* − 36074	0.9998	1.98 – 126.98	0.4337 ± 0.0117
Berberine	*y* = 41387*x* − 12535	0.9992	0.74 – 47.62	0.0952 ± 0.0067
Dehydrocorydaline	*y* = 37716*x* − 147830	0.9992	5.45 – 349.21	1.7962 ± 0.0012
Glaucine	*y* = 9473.5*x* − 9680.2	0.9990	0.69 – 44.44	1.3946 ± 0.0645
Rotundinum	*y* = 12804*x* − 5284.5	0.9999	1.5 – 96	0.5573 ± 0.0099
Corydaline	*y* = 10976*x* − 13488	0.9997	2.06 – 132.06	0.8319 ± 0.0057
Stylopine	*y* = 7135.6*x* − 12640	0.9990	0.99 – 63.56	0.6355 ± 0.0732

The linearity curves were constructed by plotting the peak area versus the concentration of each alkaloid. Each regression equation was derived from six data points (*n* = 6). For all alkaloids, the correlation coefficient *R*^2^ > 0.999, indicating that they have good linear relationship between peak area and concentration. The main alkaloids in CR crude drug were determined (*n* = 3). The alkaloids, for example, corydalmine, allocryptopine, canadine, and isocorypalmine, reported to be in CR but not being detected in our HPLC analysis were not determined.

## Data Availability

The data used to support the findings of this study are included within the article.

## Supplementary Materials

Supplementary Materials Figure S1: the cell viability of CR extracts and alkaloids. The effects of CR extracts and alkaloids in cell viability. RAW 264.7 cells in 96-well plates were treated with the different doses of CR extracts by water and different fractions of ethanol (25%, 50%, 75%, and 100%) for 24 hours, respectively. The cell viability of CR extract-treated macrophages was performed by MTT assay. The cell viability after treating CR alkaloids (dehydrocorydaline, corydaline, and corydalmine) was determined. Values are in the fold of change as compared to control, and in Mean ± SEM, *n* = 3, each with triplicate samples. ^*∗*^*p* < 0.05; ^*∗∗*^*p* < 0.01, compared to control. Graphical abstract: *Corydalis* Rhizoma (CR; the dried tuber of *Corydalis yanhusuo*) is a well-known Chinese herbal medicine. Here, the major alkaloids of CR extract were identified and evaluated in LPS-treated macrophages by determining expressions of proinflammatory cytokines, I*κ*B*α* and NF-*κ*B. It is revealed that dehydrocorydaline was the major alkaloid in CR extract, which, together with its analogous, accounted anti-inflammatory property of CR.

## References

[1] Tietge U. J. F. (2014). Hyperlipidemia and cardiovascular disease. *Current Opinion in Lipidology*.

[2] Low A. S. L., Lunt M., Mercer L. K. (2016). Association between ischemic stroke and tumor necrosis factor inhibitor therapy in patients with rheumatoid arthritis. *Arthritis and Rheumatology*.

[3] Rao P., Knaus E. E. (2008). Evolution of nonsteroidal anti-inflammatory drugs (NSAIDs): cyclooxygenase (COX) inhibition and beyond. *Journal of Pharmacy and Pharmaceutical Sciences*.

[4] Oray M., Abu Samra K., Ebrahimiadib N., Meese H., Foster C. S. (2016). Long-term side effects of glucocorticoids. *Expert Opinion on Drug Safety*.

[5] Wang Y., Fan X., Qu H., Gao X., Cheng Y. (2012). Strategies and techniques for multi-component drug design from medicinal herbs and traditional Chinese medicine. *Current Topics in Medicinal Chemistry*.

[6] Zheng X., Wu F., Lin X., Shen L., Feng Y. (2018). Developments in drug delivery of bioactive alkaloids derived from traditional Chinese medicine. *Drug Delivery*.

[7] Tang F., Tang Q., Tian Y., Fan Q., Huang Y., Tan X. (2015). Network pharmacology-based prediction of the active ingredients and potential targets of Mahuang Fuzi Xixin decoction for application to allergic rhinitis. *Journal of Ethnopharmacology*.

[8] Chen G., Xu Y., Jing J. (2017). The anti-sepsis activity of the components of Huanglian Jiedu decoction with high lipid A-binding affinity. *International Immunopharmacology*.

[9] Wang Q. S., Zhu X. N., Jiang H. L., Wang G. F., Cui Y. L. (2015). Protective effects of alginate-chitosan microspheres loaded with alkaloids from *Coptis chinensis* Franch. and *Evodia rutaecarpa* (Juss.) Benth. (Zuojin Pill) against ethanol-induced acute gastric mucosal injury in rats. *Drug Design, Development and Therapy*.

[10] Liu X., Hu Z., Shi Q. (2010). Anti-inflammatory and anti-nociceptive activities of compounds from *Tinospora sagittata* (Oliv.) Gagnep. *Archives of pharmacal research*.

[11] Zhang H., Shan Y., Wu Y. (2017). Berberine suppresses LPS-induced inflammation through modulating Sirt1/NF-*κ*B signaling pathway in RAW264.7 cells. *International Immunopharmacology*.

[12] Tang L., He Q., Meng J. Y. (2015). Rules of Ye Tian-shi’s prescriptions for treating stomachache based on data mining. *Chinese Journal of Experimental Traditional Medical Formulae*.

[13] Shen X. W., Zhang Y., Wang X. J., Zheng M. D., Zhang H. (2018). Regularity analysis of Chinese patent medicine containing Corydalis Rhizoma treating dysmenorrhea based on traditional Chinese medicine inheritance support system. *Research and Practice on Chinese Medicines*.

[14] Luo Y., Wang C.-Z., Sawadogo R., Tan T., Yuan C.-S. (2020). Effects of herbal medicines on pain management. *The American Journal of Chinese Medicine*.

[15] Qiu Z. C., Chen Y. X., Zhou R. L. (2009). Comparative study between Rhizoma Corydalis processing with vinegar and cleansing Rhizoma Corydalis in anti-inflammatory effect and analgesic effect. *Progress in Modern Biomedicine*.

[16] Matsuda H., Tokuoka K., Wu J., Shiomoto H., Kubo M. (1997). Inhibitory effects of dehydrocorydaline isolated from Corydalis Tuber against type I-IV allergic models. *Biological and Pharmaceutical Bulletin*.

[17] Xu W., Wang Y., Ma Z. (2013). L-isocorypalmine reduces behavioral sensitization and rewarding effects of cocaine in mice by acting on dopamine receptors. *Drug and Alcohol Dependence*.

[18] Yun J. (2014). L-tetrahydropalmatine inhibits methamphetamine-induced locomotor activity via regulation of 5-HT neuronal activity and dopamine D3 receptor expression. *Phytomedicine*.

[19] Yin Z. Y., Li L., Chu S. S., Sun Q., Ma Z. L., Gu X. P. (2016). Antinociceptive effects of dehydrocorydaline in mouse models of inflammatory pain involve the opioid receptor and inflammatory cytokines. *Phytomedicine*.

[20] Zhou L., Hu Y., Li C. (2018). Levo-corydalmine alleviates vincristine-induced neuropathic pain in mice by inhibiting an NF-kappa B-dependent CXCL1/CXCR2 signaling pathway. *Neuropharmacology*.

[21] Ma N. N., Li X., Jin H. (2019). Spectrum-effect relationship and mechanism of anti-inflammatory effects of different extracts of *Corydalis yanhusuo*. *Chinese Traditional and Herbal Drugs*.

[22] Wu H., Waldbauer K., Tang L. (2014). Influence of vinegar and wine processing on the alkaloid content and composition of the traditional Chinese medicine Corydalis Rhizoma (Yanhusuo). *Molecules*.

[23] Wang H., Bi F. J., In T. L., Jiang Y. Q. (2017). RP-HPLC fingerprint of *Corydalis yanhusuo* and content determination of nine alkaloids. *Journal of Chinese Medicinal Materials*.

[24] Tang Q.-Y., Zhang C.-X. (2013). Data Processing System (DPS) software with experimental design, statistical analysis and data mining developed for use in entomological research. *Insect Science*.

[25] Remichkova M., Dimitrova P., Philipov S., Ivanovska N. (2009). Toll-like receptor-mediated anti-inflammatory action of glaucine and oxoglaucine. *Fitoterapia*.

[26] Pierce J. W., Schoenleber R., Jesmok G. (1997). Novel inhibitors of cytokine-induced I*κ*B*α* phosphorylation and endothelial cell adhesion molecule expression show anti-inflammatory effectsin vivo. *Journal of Biological Chemistry*.

[27] Murphy S. F., Schaeffer A. J., Thumbikat P. (2014). Immune mediators of chronic pelvic pain syndrome. *Nature Reviews Urology*.

[28] Breser M. L., Salazar F. C., Rivero V. E., Motrich R. D. (2017). Immunological mechanisms underlying chronic pelvic pain and prostate inflammation in chronic pelvic pain syndrome. *Frontiers in Immunology*.

[29] Russo R., Cristiano C., Avagliano C. (2018). Gut-brain axis: role of lipids in the regulation of inflammation, pain and CNS diseases. *Current Medicinal Chemistry*.

[30] Li W. F., Huang H. M., Zhang Y. M. (2013). Anti-inflammatory effect of tetrahydrocoptisine from Corydalis impatiens is a function of possible inhibition of TNF-*α*, IL-6 and NO production in lipopolysaccharide-stimulated peritoneal macrophages through inhibiting NF-*κ*B activation and MAPK pathway. *European Journal of Pharmacology*.

[31] Oh Y.-C., Choi J.-G., Lee Y.-S. (2010). Tetrahydropalmatine inhibits pro-inflammatory mediators in lipopolysaccharide-stimulated THP-1 cells. *Journal of Medicinal Food*.

[32] Xiao H.-T., Peng J., Liang Y. (2011). Acetylcholinesterase inhibitors fromCorydalis yanhusuo. *Natural Product Research*.

[33] Iranshahy M., Quinn R. J., Iranshahi M. (2014). Biologically active isoquinoline alkaloids with drug-like properties from the genus *Corydalis*. *RSC Advances*.

[34] Fujii T., Mashimo M., Moriwaki Y. (2017). Expression and function of the cholinergic system in immune cells. *Frontiers in Immunology*.

[35] Borovikova L. V., Ivanova S., Zhang M. (2000). Vagus nerve stimulation attenuates the systemic inflammatory response to endotoxin. *Nature*.

[36] De Oliveira P., Gomes A. Q., Pacheco T. R., Vitorino de Almeida V., Saldanha C., Calado A. (2012). Cell-specific regulation of acetylcholinesterase expression under inflammatory conditions. *Clinical Hemorheology and Microcirculation*.

[37] Shaked I., Meerson A., Wolf Y. (2009). MicroRNA-132 potentiates cholinergic anti-inflammatory signaling by targeting acetylcholinesterase. *Immunity*.

[38] Liu E. Y. L., Xu M. L., Xia Y. (2019). Activation of G protein-coupled receptor 30 by flavonoids leads to expression of acetylcholinesterase in cultured PC12 cells. *Chemico-biological Interactions*.

[39] Kong X. P., Liu E. Y. L., Chen Z. C. (2019). Synergistic inhibition of acetylcholinesterase by alkaloids derived from stephaniae tetrandrae radix, coptidis rhizoma and phellodendri chinensis cortex. *Molecules*.

[40] Zou W., Zhang H., Bao Y. R., Meng X. S. (2012). Optimization of extraction process for Corydalis Rhizoma and correlation analysis on its chemical constituents and pharmacodynamic index. *Chinese Traditional and Herbal Drugs*.

