# Supplementary figures and images for: Dehydrocorydaline Accounts the Majority of Anti-Inflammatory Property of Corydalis Rhizoma in Cultured Macrophage
Source: Evid Based Complement Alternat Med. 2020 Nov 17;2020:4181696. doi: 10.1155/2020/4181696 (PMC7701211; doi:10.1155/2020/4181696)

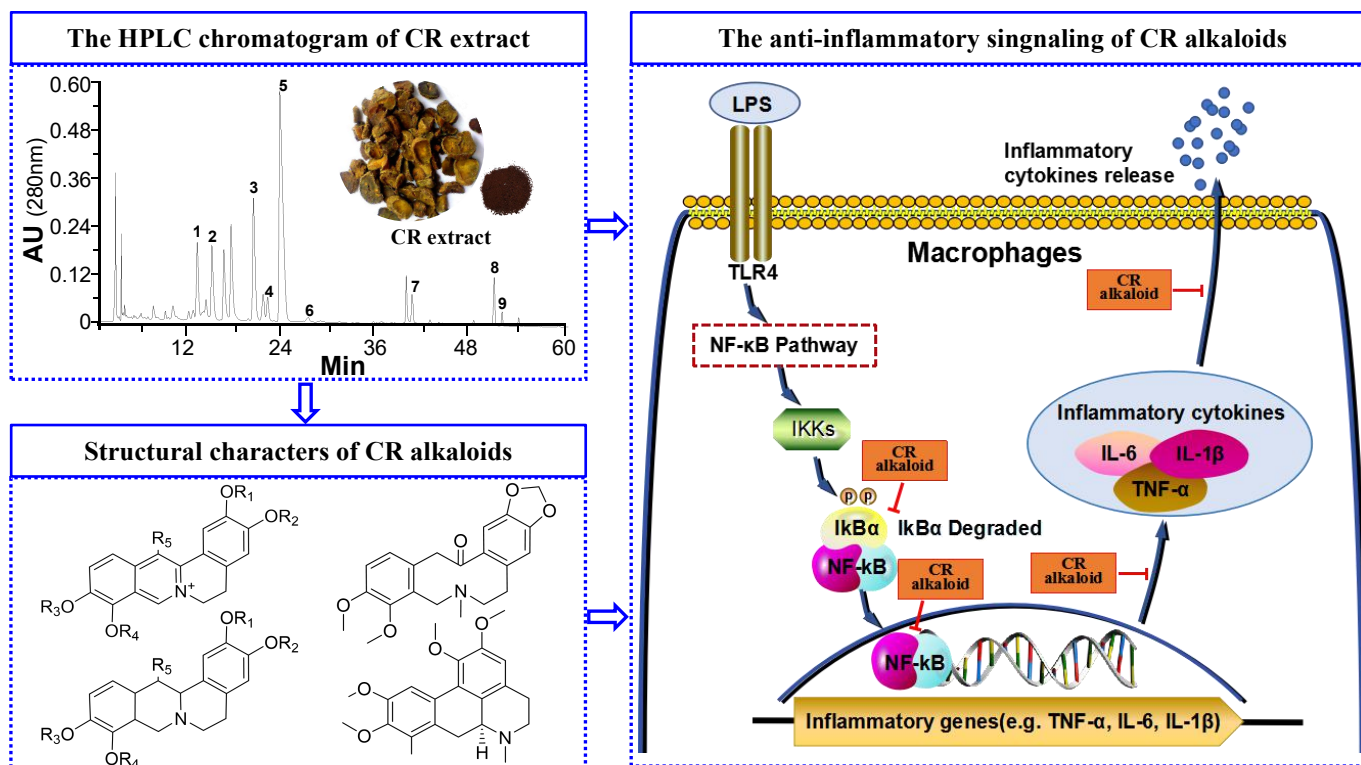

Supplement: Supplementary Materials — Figure S1: the cell viability of CR extracts and alkaloids. The effects of CR extracts and alkaloids in cell viability. RAW 264.7 cells in 96-well plates were treated with the different doses of CR extracts by water and different fractions of ethanol (25%, 50%, 75%, and 100%) for 24 hours, respectively. The cell viability of CR extract-treated macrophages was performed by MTT assay. The cell viability after treating CR alkaloids (dehydrocorydaline, corydaline, and corydalmine) was determined. Values are in the fold of change as compared to control, and in Mean ± SEM, n = 3, each with triplicate samples. ∗p < 0.05; ∗∗p < 0.01, compared to control. Graphical abstract: Corydalis Rhizoma (CR; the dried tuber of Corydalis yanhusuo) is a well-known Chinese herbal medicine. Here, the major alkaloids of CR extract were identified and evaluated in LPS-treated macrophages by determining expressions of proinflammatory cytokines, IκBα and NF-κB. It is revealed that dehydrocorydaline was the major alkaloid in CR extract, which, together with its analogous, accounted anti-inflammatory property of CR. [file 4181696.f1.zip › 4181696.f1/4181696_Graphical abstract.pdf]

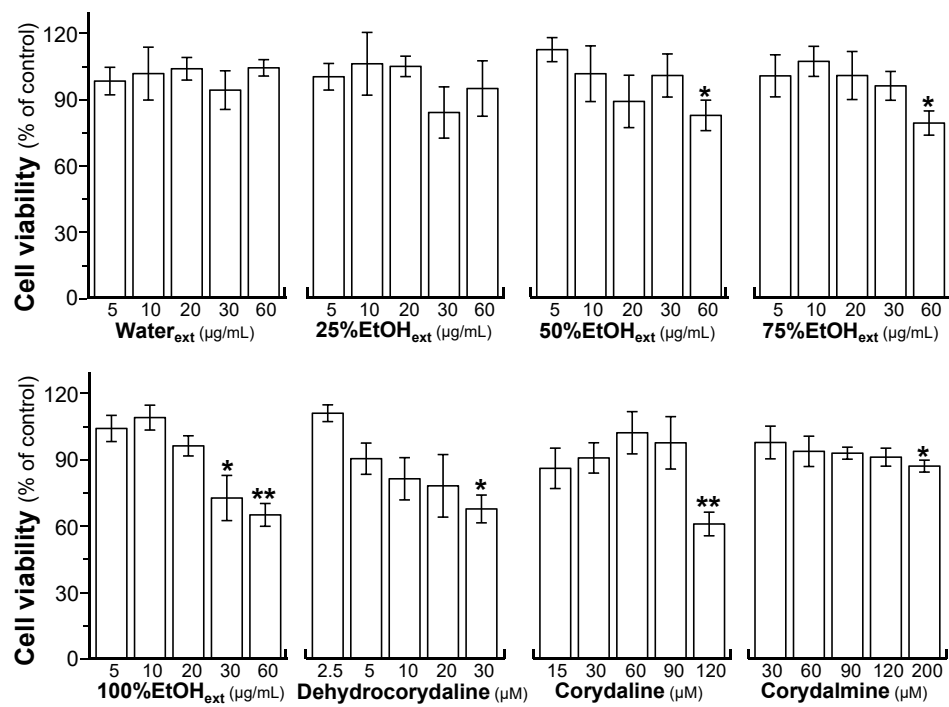

**Figure S1**  
Kong et al., 2020

Supplement: Supplementary Materials — Figure S1: the cell viability of CR extracts and alkaloids. The effects of CR extracts and alkaloids in cell viability. RAW 264.7 cells in 96-well plates were treated with the different doses of CR extracts by water and different fractions of ethanol (25%, 50%, 75%, and 100%) for 24 hours, respectively. The cell viability of CR extract-treated macrophages was performed by MTT assay. The cell viability after treating CR alkaloids (dehydrocorydaline, corydaline, and corydalmine) was determined. Values are in the fold of change as compared to control, and in Mean ± SEM, n = 3, each with triplicate samples. ∗p < 0.05; ∗∗p < 0.01, compared to control. Graphical abstract: Corydalis Rhizoma (CR; the dried tuber of Corydalis yanhusuo) is a well-known Chinese herbal medicine. Here, the major alkaloids of CR extract were identified and evaluated in LPS-treated macrophages by determining expressions of proinflammatory cytokines, IκBα and NF-κB. It is revealed that dehydrocorydaline was the major alkaloid in CR extract, which, together with its analogous, accounted anti-inflammatory property of CR. [file 4181696.f1.zip › 4181696.f1/4181696_Supplementary Figure 1.pdf]
